# A rare association between Fabry’s disease and granulomatosis with polyangiitis: a potential pathogenic link

**DOI:** 10.1186/1471-2369-15-157

**Published:** 2014-10-01

**Authors:** Hironari Hanaoka, Akinori Hashiguchi, Konosuke Konishi, Tomohiro Ishii, Masataka Kuwana

**Affiliations:** Division of Rheumatology, Department of Internal Medicine, Keio University School of Medicine, 35 Shinanomachi, Shinjuku-ku, Tokyo, 160-8582 Japan; Department of Pathology, Keio University School of Medicine, 35 Shinanomachi, Shinjuku-ku, Tokyo, 160-8582 Japan; Division of Endocrinology, Metabolism and Nephrology, Department of Internal Medicine, Keio University School of Medicine, 35 Shinanomachi, Shinjuku-ku, Tokyo, 160-8582 Japan; Department of Pediatrics, Keio University School of Medicine, 35 Shinanomachi, Shinjuku-ku, Tokyo, 160-8582 Japan

**Keywords:** Crescentic glomerulonephritis, Enzyme replacement therapy, Fabry’s disease, Granulomatosis with polyangiitis

## Abstract

**Background:**

Fabry’s disease is a rare X-linked, hereditary lysosomal storage disease caused by a deficiency of the enzyme α-galactosidase A. Granulomatosis with polyangiitis is characterized by the involvement of the respiratory tract and kidneys. Here, we report the first case of the coexistence of these diseases.

**Case presentation:**

We describe a 29-year-old man suffering from fever with maxillary sinusitis, multiple lung nodules, and proteinuria. He was diagnosed with Fabry’s disease accompanying granulomatosis with polyangiitis on the basis of the low activity of peripheral leukocyte α-galactosidase A and pathological findings in the lung and kidney. Glucocorticoid and cyclophosphamide were administered, followed by enzyme replacement therapy. Progression to end-stage renal disease has not been observed for 6 years until the time of drafting this manuscript.

**Conclusion:**

Because both Fabry’s disease and granulomatosis with polyangiitis or crescentic glomerulonephritis are rare diseases, their concurrence in this and related cases suggests there may be a pathogenic link between these two conditions. Fabry’s disease may be underdiagnosed, particularly in cases of granulomatosis with polyangiitis or crescentic glomerulonephritis.

## Background

Fabry’s disease is a rare X-linked, hereditary lysosomal storage disease caused by the deficiency of the enzyme α-galactosidase A (αGL-A). This deficiency results in the accumulation of the neutral glycosphingolipid globotriaosylceramide (GL-3) in multiple organs [1]. It causes a multisystem condition characterized by reddish-purple maculopapular lesions on the skin (angiokeratoma corporis diffusum), corneal opacities (cornea verticillata) hypohidrosis, gastroenteritis, chronic airflow obstruction, left ventricular hypertrophy, and early-onset of stroke. With advanced age, the progressive lysosomal GL-3 accumulation leads to renal failure due to the dysfunction of interstitial tubules, epithelial cells, and mesangial cells [2]. Therefore, early diagnosis of Fabry’s disease is important for successful therapeutic intervention with enzyme replacement therapy [3]. It’s diagnosis is made by demonstrating a deficiency of αGL-A in a blood sample or detection of a disease-causing mutation in the *GLA* gene. Fabry’s disease is rare. Its incidence in the United Kingdom is reported to be 0.3 per 100,000, according to the registry of all cases found between 1980 and 1995 [4, 5].

This report describes a rare case of Fabry’s disease with granulomatosis with polyangiitis (GPA), which is a multisystem inflammatory disease that affects the respiratory tract and kidneys [6]. The prevalence of GPA has increased in last 2 decades but it is still rare disease [7]. According to United Kingdom general practice research database from 1990 to 2005, it is reported to be 0.8 per 100,000 [8]. In the present case, upper and lower respiratory tract involvement and pauci-immune necrotizing and crescentic glomerulonephritis were pathologically confirmed. Glucocorticoids and oral cyclophosphamide were administered, followed by enzyme replacement therapy. Literature review found three additional cases of Fabry’s disease complicated with crescentic glomerulonephritis [9, 10]. Because both Fabry’s disease and crescentic glomerulonephritis are rare diseases, there may be a pathogenic link between these two conditions.

## Case presentation

**Figure 1:**
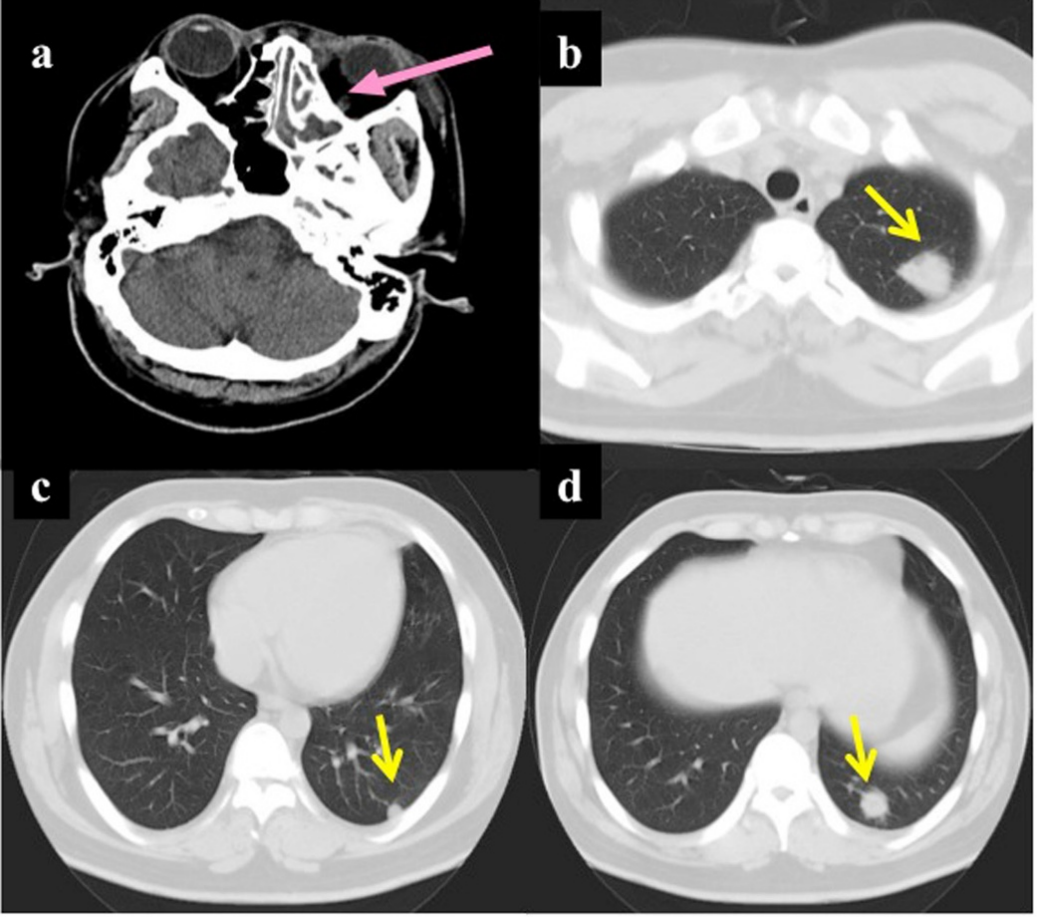
**Whole body computed tomography scan before treatment. (a)** The left nasal cavity was filled with soft tissue (pink arrow). **(b, c, d)** Multiple lung nodules were identified (yellow arrows).

**Figure 2:**
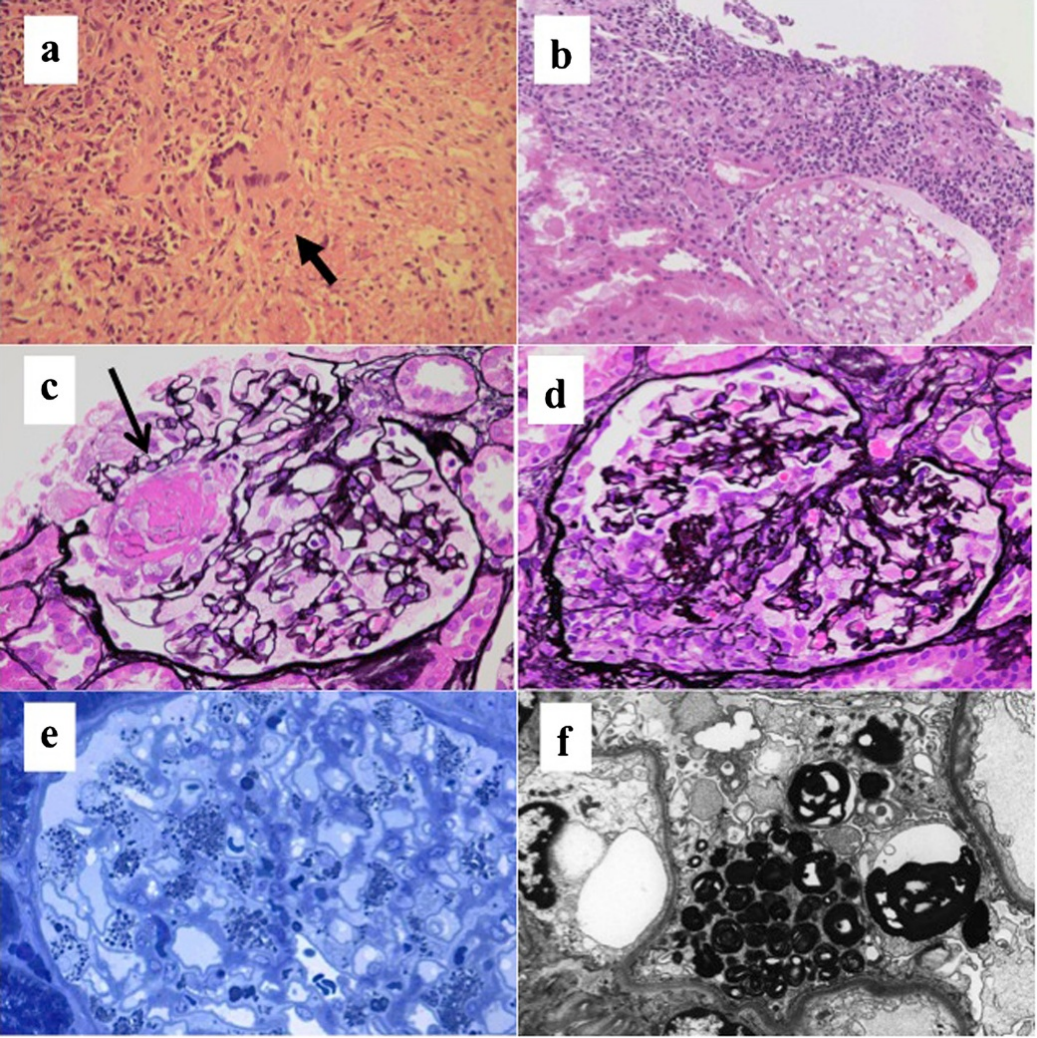
**Lung biopsy performed by computed tomography-guided needle biopsy and renal biopsy before treatment. (a)** Multi-nucleated giant cells and inflammatory cells infiltrating necrotizing lesions are shown. **(b)** Granulomatous tubulointerstitial nephritis. Hematoxylin and eosin stain, ×40. **(c)** Focal segmental necrotizing glomerulonephritis (arrow). The glomerular podocytes were swollen and vacuolated. PASM stain, ×400. **(d)** Crescentic glomerulonephritis. Podocytes in the cellular crescent were not vacuolated. PASM stain, ×400. **(e)** Inclusion bodies were detected in the podocytes. Toluidine blue stain, ×400. **(f)** Podocytes containing osmiophilic inclusion bodies ("myeloid bodies") were revealed by electron microscopy (×2000).

A 29-year-old man was admitted to our hospital on September 22, 2007 with left maxillary sinus pain and a 1-month history of general malaise and fever. His past medical history did not disclose any evidence of specific diseases, including renal diseases. So he had not taken any drugs before this admission. Family history revealed that his father died of cerebral hemorrhage at the age of 45 and his mother had no cardiovascular or renal disease. He has no sibling. At admission, the patient’s height was 174 cm, body weight was 79 kg, and his body temperature was 38.2°C. Left maxillary sinus tenderness was observed on physical examination, but no chest murmur or neurological findings were noted. A panel of laboratory studies revealed the following results: serum creatinine (Cr) 0.9 mg/dl, sodium 136.1 mEq/l, potassium 3.9 mEq/l, hemoglobin 15.1 g/dl, white blood cell count 17,600/μl, platelet count 180,000/μl, total serum proteins 6.7 g/dl, albumin 3.1 g/dl, and C-reactive protein (CRP) 16.5 mg/dl. Although most of these findings are unremarkable, the white blood cell count was marginally high, the albumin level was marginally low, and the CRP level was substantially elevated. Antinuclear antibody or myeloperoxidase-antineutrophil cytoplasmic antibody (ANCA) was not detected, but proteinase 3 (PR3)-ANCA was detected at a low titer (24 enzyme-linked immunosorbant assay unit). Results of negative serum test were obtained for Hepatitis C virus, Hepatitis B surface, and core antigens. HLA-typing was not done. Urinalysis revealed hematuria (51–100 red blood cells per high-power field) and proteinuria (0.58 g/day). Whole body computed tomography (CT) revealed left maxillary sinusitis and multiple lung nodules (Figure 1). Two-dimensional transthoracic echocardiography did not reveal signs of left ventricular hypertrophy, with an interventricular septal thickness of 10 mm and left ventricular posterior wall thickness of 10 mm. Left ventricular systolic function was preserved (ejection fraction 58.9%). A CT-guided needle biopsy of the lung showed a multinucleated giant cells and inflammatory cell infiltrate in necrotizing lesions (Figure 2a), and a renal biopsy showed focal segmental necrotizing and crescentic glomerulonephritis with interstitial granulomas. We could not find any multinucleated giant cells in renal tissue (Figure 2b–d). The glomerular podocytes were swollen and vacuolated. A semi-thin section stained with toluidine blue or electron microscopy showed numerous inclusion bodies in the podocytes (Figure 2e). Immunofluorescence revealed no IgG, IgA, IgM, C3, or C1q deposition along the capillary wall. Electron microscopy revealed lamellated inclusion bodies ("myeloid bodies") in the podocytes (Figure 2f). Analysis of the leukocytes demonstrated an αGL-A activity level of 11.8 nmol/h/mg (normal range: 49.6–116 nmol/h/mg). After careful consideration of the findings, although this patient lacked any other pathognomonic signs of Fabry’s disease, such as acroparesthesias, dyshidrosis, or cutaneous angiokeratomas, a diagnosis of Fabry’s disease associated with GPA was made.

Treatment with prednisolone (PSL, 60 mg/day) and oral cyclophosphamide (100 mg/day) was initiated on October 6, 2007. The patient received enzyme replacement therapy in the form of agalsidase alfa at 0.2 mg/kg every other week starting July 14, 2008. Complete resolution of the proteinuria, lung nodules, and maxillary sinusitis was seen within 2 months of initiation of this therapy. Oral cyclophosphamide was discontinued January 27, 2009, and azathioprine (50 mg/day) was started for maintenance therapy. The PSL dosage was gradually tapered to 9 mg/day by August 2009. At last follow-up on October 16, 2013, the patient’s Cr was 1.3 mg/dl.

## Discussion

This is the first report of the coexistence of Fabry’s disease and GPA, although Fabry’s disease with necrotizing crescentic glomerulonephritis has been reported in 3 cases previously [9, 10]. We reviewed the three previous cases and compared their findings with those of the present case (Table 1). Interestingly, none of the previous cases were diagnosed as Fabry’s disease before a diagnosis of renal disease was made. Pauci-immune glomerulonephritis was confirmed in the present case and in the two cases reported by Singh, but the remaining patient demonstrated IgG-positive and C3-positive immune deposits along the capillary wall. Serum Cr was increased in the 3 previous cases (1.4–4.7 mg/dl) but not in the present case (0.9 mg/dl), and the amount of proteinuria in the present case was relatively low (0.6–1.3 g/day). Only the present case was serologically positive for PR3-ANCA and definitively diagnosed as GPA. Low peripheral leukocyte αGL-A activity was detected in all three males, but not in the heterozygous female. All four patients were treated with high-dosage prednisolone, and cyclophosphamide was added in three cases. No deaths have been reported and relatively benign courses in short observation periods have been reported. Because renal pathological findings, including the coexistence of granulomatous tubulointerstitial nephritis, were not shown in detail and a survey of the upper and lower respiratory tracts was not done in the three previous cases, the coincidence of subclinical GPA cannot be precisely confirmed.

**Table 1:** **Demographic and clinical features of patients with Fabry’s disease complicated by crescentic glomerulonephritis**

	Singh et al. (5)	Singh et al. (5)	Shimazu et al. (6)	This case
**Age (years)**	11	26	58	29
**Gender**	M	F	F	M
**Chief complaint**	Fever, arthralgia	Fever, pedal edema	Hematuria, pedal edema	Fever, sinus tenderness
**BUN (mg/dl)**	35	ND	42.3	14.4
**Cr (mg/dl)**	2.6	1.4	4.7	0.9
**Proteinuria** **(g/day)**	1.3	0.7	ND	0.6
**PR3 ANCA**	Negative	Negative	Negative	Positive
**MPO ANCA**	Negative	Negative	Negative	Negative
**Anti-GBM antibody**	Negative	Negative	Negative	Negative
**ANA**	Negative	Negative	Negative	Negative
**Pauci-immune response**	Yes	Yes	No	Yes
**Diagnosis of GPA**	No	No	No	Yes
**αGL-A activity**	< 10%^*1^	50%^*^	76.1^**^	11.8^***^
**Mutation in the αGL-A gene**	ND	ND	ND	p.N224H/c.670A > C
**Treatment**	PSL IVCY	PSL CYC 150 mg/day	PSL 60 mg/day HD	PSL 60 mg/day CYC 100 mg/day
**Follow-up period (months)**	16	15	3	72
**Final Cr level (mg/dl)**	8.4	1.34	2.0–3.0	1.30

^*1^Percentage of normal mean is shown, ^**^hetero normal range is 5–100 nmol/h/mg, ^***^normal range is 49.6–116 nmol/h/mg. ANA, antinuclear antibody, ND: not determined, GPA: granulomatosis with polyangiitis, PSL: prednisolone, IVCY: intravenous cyclophosphamide, CYC: oral cyclophosphamide, HD: hemodialysis, αGL-A: α-galactosidase A.

The pathogenic relationship between Fabry’s disease and GPA remains unknown. Galactocerebroside that accumulates in Fabry’s disease may be immunogenic [11], leading to the emergence of immune-mediated disease processes. Recently, Lin et al. demonstrated that neutrophil apoptosis in acute lung injury is inhibited via sphingolipid signaling [12]. Neutrophils play the primary pathogenic role in GPA [13–15]. Priming by proinflammatory cytokines causes translocation of ANCA antigens from the lysosomal compartments of neutrophils to the cell surface during the early phase of the inflammatory process. Engagement of ANCA with their antigens on the cell surface and interaction of the Fc part of the antibody with Fc receptors activate the neutrophils. Activated neutrophils adhere to vessel walls, produce reactive oxygen radicals, and release lysosomal enzymes, ultimately resulting in necrotizing vascular injury. Accumulated glycosphingolipid may provide long-term survival for neutrophils and contribute to their sustained activation, exacerbating vascular injury.

Recently, a renal variant of Fabry’s disease that solely affects kidneys was recognized in hemodialysis patients [16]. Nakao et al. reported that 6 (1.2%) out of 514 hemodialysis patients had low plasma αGL-A activity. This finding suggests that Fabry’s disease may be underdiagnosed in general and may occur more often than expected in patients with GPA or crescentic glomerulonephritis. Low αGL-A activity may not result from an inherited genetic mutation; it may reflect individual variation in its production or regulation. Further studies will be required to investigate the pathogenic link between the two diseases.

## Conclusions

We describe a case in which Fabry’s disease and granulomatosis with polyangiitis occurred together, and discuss three additional cases in which Fabry’s disease has been reported to be concomitant crescentic glomerulonephritis. The remarkable concurrence of these disorders in this number of cases suggests these two diseases may share a pathogenic link. The information presented in this report suggest that Fabry’s disease may, in fact, be underdiagnosed, particularly in cases of GPA or crescentic glomerulonephritis.

## Consent

Written informed consent was obtained from the patient for publication of this Case report. A copy of the written consent is available for review by the Editor of this journal.

## Abbreviations

αGL-A: α-galactosidase A
ANCA: Antineutrophil cytoplasmic antibody
CRP: C-reactive protein
CT: Computed tomography
GL-3: Globotriaosylceramide
GPA: Granulomatosis with polyangiitis
PR3: Proteinase 3
PSL: Prednisolone.
